# Multiphysics Modeling of Electrochemical Impedance Spectroscopy Responses of SAM-Modified Screen-Printed Electrodes

**DOI:** 10.3390/s24030858

**Published:** 2024-01-28

**Authors:** Lara Franchin, Stefano Bonaldo

**Affiliations:** Department of Information Engineering, University of Padova, 35131 Padova, Italy; stefano.bonaldo@unipd.it

**Keywords:** multiphysics model, self-assembled monolayer, screen-printed electrodes, electrochemical impedance spectroscopy

## Abstract

In this work, we present a multiphysics modeling approach capable of simulating electrochemical impedance spectroscopy (EIS) responses of screen-printed electrodes (SPEs) modified with self-assembled monolayers of 11-Mercaptoundecanoic acid (MUA). Commercially available gold SPEs are electrochemically characterized through experimental cyclic voltammetry and EIS measurements with 10 mM [Fe(CN)_6_]^3−/4−^ redox couple in phosphate buffered saline before and after the surface immobilization of MUA at different concentrations. We design the multiphysics model through COMSOL Multiphysics^®^ based on the 3D geometry of the devices under test. The model includes four different physics considering the metal/solution interface electrochemical phenomena, the ion and electron potentials and currents, and the measurement set-up. The model is calibrated through a set of experimental measurements, allowing the tuning of the parameters used by the model. We use the calibrated model to simulate the EIS response of MUA-modified SPEs, comparing the results with experimental data. The simulations fit the experimental curves well, following the variation of MUA concentration on the surface from 1 µM to 100 µM. The EIS parameters, retrieved through a CPE-modified Randles’ circuit, confirm the consistency with the experimental data. Notably, the simulated surface coverage estimates and the variation of charge transfer resistance due to MUA-immobilization are well matched with their experimental counterparts, reporting only a 2% difference and being consistent with the experimental electrochemical behavior of the SPEs.

## 1. Introduction

The development of electrochemical biosensors has attracted considerable interest in the last years due to their cost-effective highly-sensitive detection and their inexpensiveness [1,2,3]. Electrochemical biosensors are employed in a wide range of applications, e.g., smart agriculture and sustainable food production [4,5,6,7,8] and clinical diagnostics and smart healthcare [9,10,11,12,13,14]. For instance, the authors in [7] report an innovative biosensor based on bacterial proliferation for the timely detection of phages in milk samples, which is an important requirement for food safety in dairy production. In [13], the authors show a highly sensitive and selective biosensor for the detection of tau protein, a biomarker for early diagnosis of Alzheimer’s disease. Another valuable example is reported in [14], where a flexible graphene oxide sensor with a detection limit of 0.91 µM is developed to detect the presence of H_2_O_2_ as the biomarker for a wide number of pathologies, such as Alzheimer’s, diabetes, cancer, brain injury, and neurodegenerative disorders. The design of biosensor devices, despite proving to be powerful resources exploitable in various sectors, frequently relies on empirical assessments of their performance rather than substantial insights into their modeling before fabrication. Engineering and nanotechnology heavily rely on finite-element simulations to mathematically model physical systems. The modeling facilitates the exploration of various designs and parametric configurations within the system, enabling the assessment of expected behavior and outcomes through alterations such as geometries, surface morphologies, properties, and testing conditions. COMSOL Multiphysics^®^ is a commercial finite-element software which allows multiphysics simulations of non-standard geometries combining a wide range of physical phenomena, e.g., hydrodynamic and electrochemical processes [15]. The complexity of modeling the electrical responses of biosensors, like cyclic voltammetry (CV) and electrochemical impedance spectroscopy (EIS), arises from the necessity to integrate synergistic physics in the model, with appropriately calibrated parameters associated with device materials and testing conditions, employing specific equivalent circuits for signal generation and conditioning—such as potentiostat for CVs and frequency response analyzers for EISs. Finite-element simulations in COMSOL Multiphysics^®^ can be exploited to design optimized electrodes [15,16,17,18,19,20], studying their electrochemical behavior to develop biosensors with enhanced performance. In [20], for example, an o-quinone enzymatic biosensor was modeled through COMSOL Multiphysics^®^ and validated experimentally to study and optimize its detection capability. Despite the advantages, there are still a limited number of studies that document sensor modeling, often lacking validation through experimental measurements [16,17], seldom considering the full electrode characterization, and not thoughtfully investigating the physical, chemical, and electrical phenomena on its surface.

In this work, we present a multiphysics model capable of simulating EIS responses of electrochemical devices in the presence of surface self-assembled monolayer (SAM) functionalization. The simulations are carried out with a refined 3D multiphysics model designed with COMSOL Multiphysics^®^ and based on screen-printed electrodes (SPEs). To ensure accurate prediction of experimental outcomes, our model undergoes meticulous calibration using a series of experimental CV and EIS measurements at different concentration levels of SAM on the surface. Subsequently, we assess the model’s effectiveness by accurately simulating an additional set of EIS experimental measurements of varying surface functionalization through the immobilization of self-assembled monolayers.

## 2. Materials and Methods

### 2.1. Electrodes under Evaluation and Measurements Set-Up

**Figure 1:**
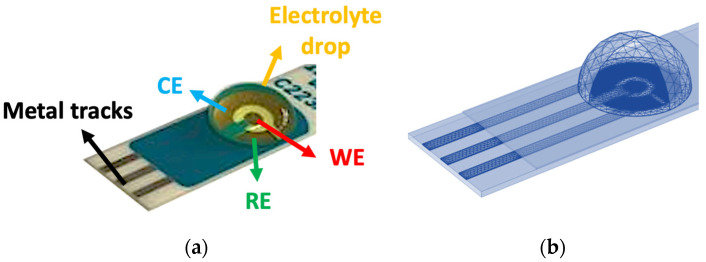
(**a**) Layout of the devices under evaluation. (**b**) 3D geometrical structure and mesh of the model implemented in COMSOL Multiphysics^®^.

The devices under evaluation were commercially available low-cost screen-printed electrodes (DRP223BT, Metrohm DropSens, Spain). These sensors were constructed with a conventional three-electrode setup, featuring gold (Au) electrodes for both the working (WE) and counter (CE) electrodes along with a silver (Ag) reference electrode (RE), all printed on a ceramic substrate measuring 34 mm × 10 mm × 0.5 mm. Figure 1a illustrates the geometrical arrangement, showcasing a central disk of 1.6-mm diameter as the WE. The electrolyte solution is placed as a 100-µL drop on top of the electrode area.

For experimental characterization, the SPEs underwent analysis through cyclic voltammetry (CV) and electrochemical impedance spectroscopy (EIS) using a 100 µL solution of Fe(CN)_6_^3−^/Fe(CN)_6_^4−^ (referred as FeCN) at 10 mM of concentration in 1 × Phosphate Buffered Saline (PBS). Employing the Solartron 1260 electrochemical impedance analyzer and the Solartron 1287 electrochemical potentiostat, CV measurements were conducted at a scan rate of 100 mV/s. For bare SPEs, the potential sweeps ranged from −0.2 V to 0.45 V, whereas for SAM-modified SPEs, they extended from −0.8 V to 0.8 V. The internal Ag electrode was used as a pseudo-reference. The redox equilibrium potential *E_0_* was deduced from the CV curves, yielding *E_0_* = 132 mV for bare electrodes and *E_0_* = 110 mV for SAM-modified devices. EIS measurements, carried out at *V_DC_* = *E_0_* and *V_ac_* = 10 mV, spanned a frequency range between 1 Hz and 100 kHz, using a 3-electrode configuration to characterize only WE, isolating its contributions from other electrodes [21,22]. All experimental measurements were replicated a minimum of 3 times using different devices. The EIS electric parameters were extrapolated through a modified Randles’ circuit. The circuit comprises a faradaic branch, composed by a series of a charge transfer resistance (*R_ct_*) and a Warburg element (*W*), and a parallel non-faradaic contribution where the double layer capacitance (*C_dl_*) is substituted by a constant phase element (*CPE*). The parallel is in series with the solution/electrode resistance contribution (*R_s_*).

### 2.2. Multiphysics 3D Model

The COMSOL Multiphysics^®^ simulator operates on the finite-element method, enabling the integration of various equations governing diverse physical phenomena [23]. Our proposed model is structured in a 3D geometry, where each element possesses distinct material properties. This geometric structure comprises components like the substrate, working, counter, and reference electrodes (each 100 µm thick), metal interconnections and contact areas, an isolation layer, and the solution drop—a semi-sphere with a radius of 4.5 mm. Presently, the simulator doesn’t incorporate surface roughness.

The 3D layout, involving elements like the solution drop, electrodes, and metal interconnections, is meticulously meshed using tetrahedral elements (Figure 1b). This mesh is especially refined around the interfaces of electrodes with the metal/solution, enhancing accuracy in describing electrochemical reactions. The first layer thickness of 1 µm is considered the diffusive layer, with a stretching factor of 1.1 in the volume. Simulations run within a reasonable computational timeframe—approximately 1 h for each CV curve and about 7 min for the entire frequency sweep from 100 kHz to 1 Hz for every EIS response.

The model incorporates four distinct COMSOL physics [24], enabling precise simulation of microscopic mechanisms that combine electrochemical, electrical, and chemical phenomena. These include the distribution of electrochemical species in the solution and the current exchange at the electrode/solution interface. Equations defining these processes are applied to specific elements within the 3D geometry. These equations undergo evaluation and resolution within a time-dependent transitory study. The electrochemical reaction kinetics at metal/solution interface and the electric currents and potentials applied to the electrodes and the electrolyte are modeled through the *secondary current distribution physics*. The physics is based on the extended Butler–Volmer equation where *C_R_* and *C_O_* are the redox species concentrations, *j_0_* is the exchange current density, *α_c_* and *α_a_ = (1* − *α_c_)* are redox symmetry factors, and *η = Φ_s_* − *Φ_l_* − *E_eq_* is the overpotential; where *Φ_s_* is the electrode potential, *Φ_l_* is the electrolyte potential, and *E_eq_* is the equilibrium potential of the electrochemical cell, retrieved from *E_0_* using the Nernst equation for a one-electron process.

(1) $$j=j_{0}\left[C_{R}\exp\left(\frac{\alpha_{a}F\eta }{RT}\right)-C_{O}\exp\left(\frac{{-\alpha }_{c}F\eta }{RT}\right)\right],$$

The diffusive transport and the reactions of the redox couple species in the electrolyte solution are simulated through *transport of diluted species physics*, which implements the Nernst–Planck equation coupled with *secondary current distribution physics*, assuming a prevalence of diffusive mass transport. The current distributions and the electric potential on the metal tracks are modeled through *electric current physics*, based on Maxwell’s classical laws, assuming negligible inductive effects. Finally, the electric signals generation, conditioning, and readouts of an ideal potentiostat are modeled through the *electric circuit module*, coupled with the metal contacts area. The ideal potentiostat is simulated through an equivalent circuit with two operational amplifiers (gain of 10^5^) which applies a linear sweep tension to control the potential between WE and RE and reads the current between WE and CE.

## 3. Results and Discussion

### 3.1. Bare SPEs Model Calibration

**Figure 2:**
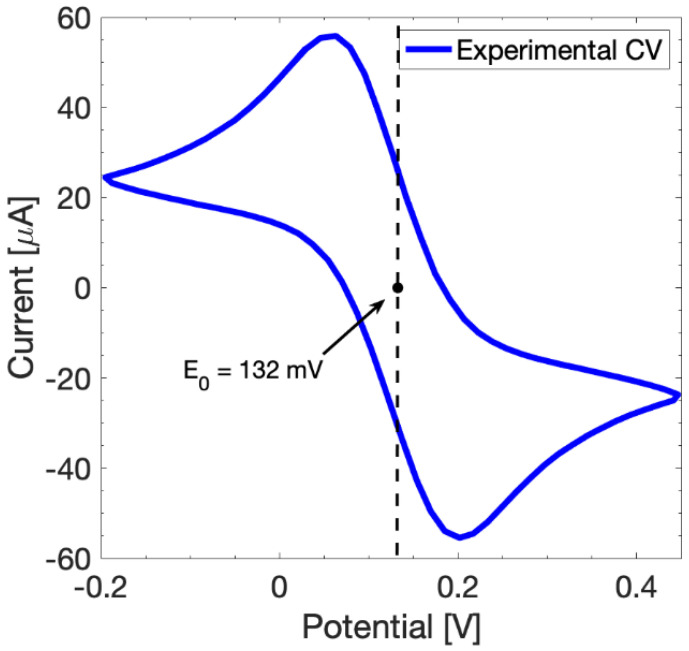
CV response at scan rate of 100 mV/s in a potential range from −0.2 V to 0.45 V of bare SPEs to retrieve the *E_0_*.

The proposed model is calibrated through the definition of key parameters in order to get a good fit between simulations and experimental results. The key parameters are extrapolated from a dedicated set of experimental CV and EIS measurements performed in a solution of 10 mM Fe(CN)_6_^3−^/Fe(CN)_6_^4−^ in PBS. The redox equilibrium potential *E_0_* is retrieved from the CV response as well as the charge transfer coefficients *α_a_* and *α_c_*, estimated by evaluating the symmetry of the CV curve (Figure 2).

While a *CPE* could be more suitable to model the double layer of experimental electrochemical responses, to our knowledge the used software does not allow the consideration of a frequency-dependent value to model the double layer. Hence, we have estimated the double layer capacitance *C_dl_* from the *CPE*. The impedance of the constant phase element (*Z_CPE_*) is defined as

(2) $$Z_{CPE}=\frac{1}{Y_{0}{\left(i\omega \right)}^{n}},$$

where *ω* is angular frequency and *Y_0_* and *n* are the characteristic parameters of the *CPE*.

Since the parameter *Y_0_* does not have the same physical meaning as a capacitance, it is fundamental to apply a conversion method to obtain an equivalent *C_dl_* value from the *CPE*. Numerous approaches have been reported in literature [25] and we choose to estimate the *C_dl_* value as shown in [26]:

(3) $$C_{dl}=Y_{0}{\left(\omega_{max}^{\prime \prime }\right)}^{n-1},$$

where ω″_max_ is the frequency at which the impedance imaginary part is at its maximum.

Meanwhile, the exchange charge density current *j_0_* is estimated from charge transfer resistance *R_ct_* through (4) [27,28].

(4) $$j_{0}=\frac{RT}{nFA\;R_{ct}},$$

Both *CPE* and *R_ct_* values are extrapolated through fitting the EIS curve with the modified Randles’ equivalent circuit. The redox species diffusion coefficients *D_O_* and *D_R_* are retrieved from literature [29,30], and they are considered identical, being approximated to the nearest integer. Table 1 reports the used estimated values of the key parameters.

**Figure 3:**
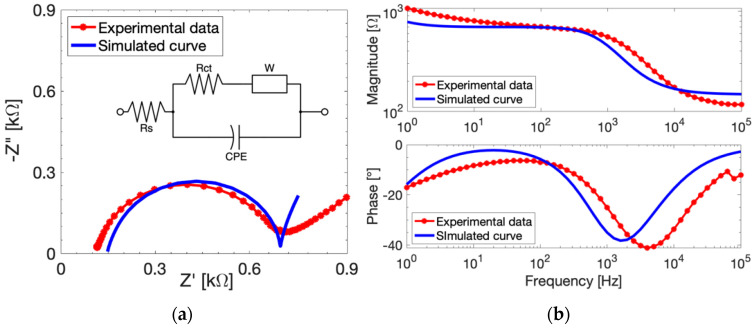
Experimental (red dotted curves) and simulated (blue curves) electrochemical responses of bare electrodes measured with 10 mM FeCN in PBS. (**a**) EIS Nyquist diagram at *V_DC_* = 132 mV and *V_AC_* = 10 mV, with CPE-modified equivalent circuit as inset. (**b**) Magnitude (**top**) and phase (**bottom**) of EIS responses at *V_DC_* = 132 mV and *V_ac_* = 10 mV.

After the model parameters extrapolation, we simulate the EIS responses of bare gold electrodes with COMSOL, imposing the same experimental conditions of the real measurements. The simulated curves are compared to their experimental counterparts in Figure 3. The simulated EIS Nyquist plot (Figure 3a) follows the trend of the experimental curve until the Warburg branch, where the diffusivity and other non-modellable slow processes start to influence the experimental measurements. Overall, the model is able to predict the EIS measurements quite well. The EIS parameters are extrapolated through the CPE-modified Randles’ circuit reported as an inset in Figure 3a. The simulated *R_ct_* value is only less than 15% different from the experimental value. The Bode magnitude diagram (Figure 3b, top plot) evidences a slight increase of the simulated *Rs* with respect to the experimental value (+27.5%). This difference can be attributed to the experimental error since the simulator accounts for ideal conditions. Meanwhile, the Bode phase plot (Figure 3b, bottom) shows as phase displacement of the simulated curve due to shift at an intermediate frequency around 2 kHz. It is worth noting that COMSOL does not allow the simulation of a constant phase element and accounts only for double layer capacitance. The *C_dl_* value used in the simulation needs to be estimated from *CPE* with (3), as previously reported, partially affecting the accuracy of the model estimate. In addition, the simulated curve can be fitted with the equivalent electric circuit with a *CPE* parameter *n* equal to 1, demonstrating that the model implements an ideal capacitance as the double layer. On the other hand, the experimental data show *n =* 0.91, evidencing that, experimentally, the double layer should be considered as almost-ideal capacitance. These considerations are most likely the cause of frequency shift visible in Figure 3b at low-intermediate frequencies.

### 3.2. SAM-Modified SPEs Model Calibration

We functionalize the SPEs’ surface with a SAM of 11-Mercaptoundecanoic acid (MUA). We selected MUA due to its strong binding with gold surfaces and the stability of its monolayer. In addition, MUA is widely used for surface passivation before immobilizing specific recognition elements such as proteins and antibodies [31,32]. The electrodes are soaked in an ethanolic solution at different concentrations of MUA in a range from 1 µM to 100 µM for 1 h to allow for the SAM’s immobilization. The time of functionalization and the concentration range are retrieved from well-consolidated immobilization procedures reported in the literature [32]. We carefully rinsed the devices with bi-distilled water and then perform electrochemical characterizations through CV and EIS measurements with a concentration of 10 mM FeCN in PBS. We take as reference the CV and EIS measurements with SPEs functionalized with MUA at a concentration of 1 µM to report the calibration results.

**Figure 4:**
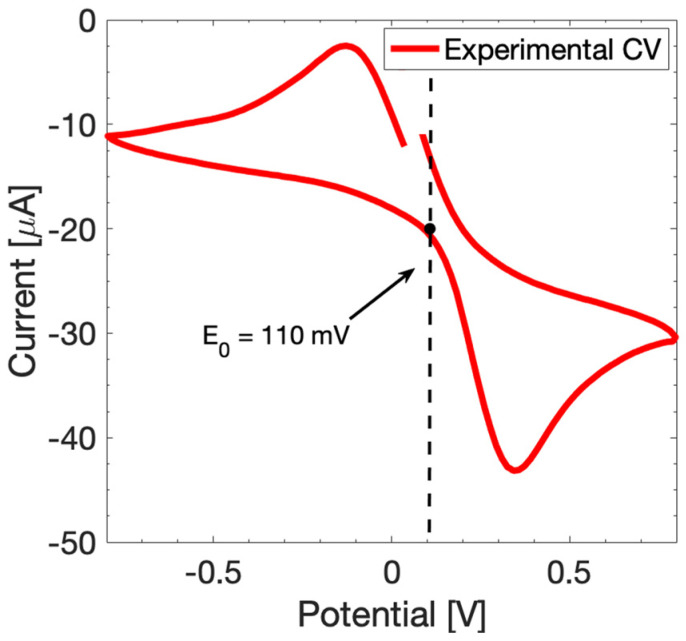
CV response at scan rate of 100 mV/s in a potential range from −0.8 V to 0.8 V of 1 µM-MUA-modified SPEs to retrieve the *E_0_*.

To simulate the electrochemical response of MUA-modified SPEs, we need to take into account the SAM surface modification in the proposed model. The molecular immobilization can be modeled through a variation of the exchange current density *j_0_*, caused by the change in the electron transfer capabilities due to the surface modification, which reflects on the parameter [33,34]. Hence, the exchange current density *j_0_* at each considered MUA concentration is calculated once again through (2) by extrapolating the *R_ct_* values from experimental EIS curves. The redox equilibrium potential *E_0_* is retrieved from the CV response as well as the charge transfer coefficients *α_a_* and *α_c_*, estimated by evaluating the symmetry of the CV curve (Figure 4). In this case, the retrieved value of *E_0_* is 110 mV, while the current peaks present a slight asymmetry since the anodic and cathodic peaks ratio is 0.8. Therefore, the symmetry coefficients are adjusted accordingly. *C_dl_*, *D_ox_*, and *D_red_* are retrieved as reported in Section 3.1. The values used for the calibration of the 1 µM-MUA-modified SPEs simulation are reported in Table 2.

**Figure 5:**
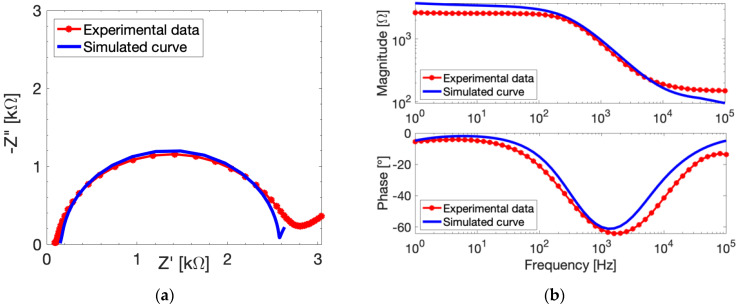
Experimental (red dotted curves) and simulated (blue curves) electrochemical responses of 1 µM-MUA-modified SPEs measured with 10 mM FeCN in PBS. (**a**) EIS Nyquist diagram at *V_DC_* = 132 mV and *V_AC_* = 10 mV. (**b**) Magnitude (**top**) and phase (**bottom**) of EIS responses at *V_DC_* = 132 mV and *V_ac_* = 10 mV.

After the parameters’ extrapolation, we simulated the EIS responses of MUA-modified gold electrodes with COMSOL imposing the same experimental conditions of the real measurements. The simulated and the experimental EIS signals of 1 µM-MUA-modified SPEs are compared in Figure 5. The experimental EIS Nyquist plot (Figure 5a) is well-matched with the simulated curve, reporting only a slight difference at the Warburg branch, characterized by slow diffusive processes at lower frequencies which are highly affected by experimental error. The *R_ct_* values are extrapolated with the CPE-modified Randles’ circuit, and they differ only by 7%. The simulated *Rs* is 40-Ω higher with respect to the experimental value, which is evident in the Bode magnitude diagram (Figure 5b, top plot). This difference between the simulation and the experimental behavior is possibly caused by a non-homogenous functionalization of the surface due to non-ideality of the real device. Moreover, the Bode phase plot (Figure 5b, bottom) reports a displacement at the high-intermediate frequencies shown—similar to the bare electrodes—which is once again probably due to the double layer capacitance. An interesting aspect is that the frequency shift in Figure 5b is not as evident as Figure 3b. This is probably due to the surface functionalization. In this case, the experimental *CPE* parameter *n* is equal to 0.95, which is higher that the bare electrode *n* value, suggesting that the experimental double layer may be closer to an ideal capacitance in the presence of surface functionalization.

**Figure 6:**
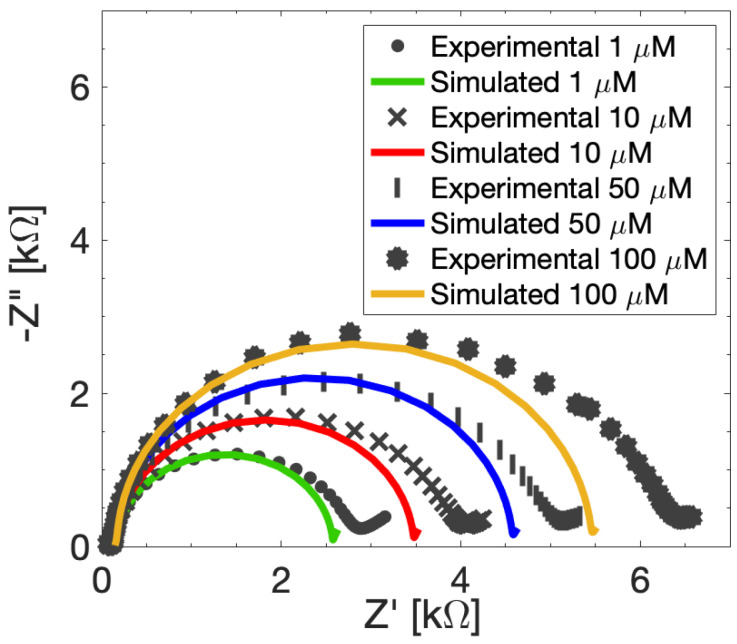
Experimental (red dotted curves) and simulated (blue curves) EIS Nyquist diagram of the electrochemical responses of modified SPEs at different MUA concentrations measured with 10 mM FeCN in PBS. at *V_DC_* = 132 mV and *V_AC_* = 10 mV.

After model calibration, we simulate a new data set of experimental measurements at different MUA concentrations, aiming to demonstrate the model’s predictive capability of EIS response for SAM-modified SPEs with different levels of surface functionalization. In this case, all the model parameters are kept fixed except for *j_0_*, which is adjusted according to the MUA concentration used for the functionalization. Figure 6 reports the comparison between the simulations and the Nyquist plots of the experimental EIS measurements with different MUA concentrations. All the simulated curves fit their experimental counterparts well from high frequencies to medium-low frequencies (100 kHz to 100 Hz) and are able to follow the increase of MUA functionalization on the surface with the same considerations as discussed above. In particular, the simulated *R_ct_* are well matched with the experimental values, e.g., for an MUA concentration of 50 µM the variation between simulated and experimental *R_ct_* is less than 5%. Therefore, it can be concluded that the proposed model is capable of simulating EIS responses of SPEs accounting for MUA presence at different concentrations.

### 3.3. Assessment of Simulated Surface Coverage and Variation of Charge Transfer Resistance

In electrochemical biosensing applications, the variation of the charge transfer resistance is often a crucial parameter to determining whether a surface modification has occurred correctly or if the device under examination can detect the analytes of interest. Having a model capable of simulating this behavior can be helpful for sensors design and development before actual device fabrication. Hence, in this section we evaluate how the proposed model can predict the experimental variation of charge transfer resistance due to the surface functionalization. First, we calculate the level of the molecular immobilization on the electrode by calculating the surface coverage *θ* using (4) through the *R_ct_* values of bare electrodes and MUA-functionalized devices, extrapolated from EIS experimental measurements [35].

(5) $$\theta =1-\frac{R_{ct\_bare}}{R_{ct\_MUA}}$$

**Figure 7:**
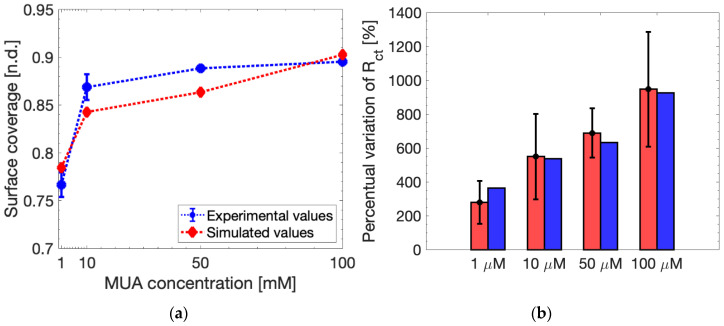
(**a**) Simulated surface coverage compared to the experimental data. (**b**) Simulated *R_ct_* variation to the experimental data measured at *V_DC_* = 132 mV and *V_ac_* = 10 mV.

The comparison between the simulated and experimental *θ* is reported in Figure 7a. It is evident that the simulated data follow the same trend as the experimental values, and the reported coverage only differs by <2% for all the MUA concentrations. Hence, simulated surface coverage is very similar to the experimental *θ*. The percentual variation of the charge transfer resistance (Δ*R_ct_*) between MUA-modified sensors and their bare measurements is evaluated in Figure 7b. All the simulated Δ*R_ct_* show a good match with the experimental average values, being well within the reported experimental error for all the MUA-concentrations. These results clearly demonstrate the model capability to predict the electrochemical response of SPEs devices after surface functionalization with MUA, suggesting that the proposed model may also be a reliable tool for simulating electrochemical detection of other surface-immobilized molecules such as DNA and proteins.

## 4. Conclusions

In this work, we propose a multiphysics model to simulate the electrochemical responses of MUA-functionalized commercial screen-printed biosensors. The model requires a careful calibration through dedicated tests with CV and EIS measurements. The model is validated by simulating a new set of EIS curves at different MUA concentrations. Our model has proven to be a reliable tool for the simulation and prediction of the electrochemical behavior of biosensing systems with surface molecular immobilization, being useful for simulating the electrochemical response of functionalized screen-printed electrodes, which may aid the development and design of biosensors before device fabrication. Simulated curves of MUA-modified electrodes fit the experimental data well, with simulated *R_ct_* < 7% different from the experimental values. The variation of *R_ct_* due to MUA surface immobilization at different concentrations reports values well within the experimental error, and the simulated surface coverage is only 2% less than the experimental values. These promising results suggest that the model can be exploited to simulate a wide range of biosensing applications, including more complex surface functionalization. In the future, we plan to further test the model considering DNA immobilization and protein presence on the surface device.

## Figures and Tables

**Table 1:** List of model key parameters used to calibrate the model for bare electrodes.

Parameter	Value
*E_0_*	132 [mV]
*C_dl_*	0.1 [F/m^2^]
*j_0_*	2.5 [A/m^2^]
*D_R_ = D_O_*	7 × 10^−6^ [cm^2^/s]
*α_a_*	0.5
*α_c_*	0.5

**Table 2:** List of parameters used to calibrate the model for SAM-modified electrodes.

Parameter	Value
*E_0_*	110 [mV]
*C_dl_*	0.1 [F/m^2^]
*j_0_*	0.55 [A/m^2^]
*D_red_ = D_o_*	7.1 × 10^−6^ [cm/s]
*α_a_*	0.6
*α_c_*	0.4

## Data Availability

Data are contained within the article.
